# Goats (*Capra hircus*) From Different Selection Lines Differ in Their Behavioural Flexibility

**DOI:** 10.3389/fpsyg.2021.796464

**Published:** 2022-02-01

**Authors:** Christian Nawroth, Katrina Rosenberger, Nina M. Keil, Jan Langbein

**Affiliations:** ^1^Research Institute for Farm Animal Biology, Institute of Behavioural Physiology, Dummerstorf, Germany; ^2^Swiss Food Safety and Veterinary Office, Centre for Proper Housing of Ruminants and Pigs, Agroscope Tanikon, Ettenhausen, Switzerland

**Keywords:** animal cognition, discrimination learning, farm animals, reversal learning, ungulates

## Abstract

Given that domestication provided animals with more stable environmental conditions, artificial selection by humans has likely affected animals' ability to learn novel contingencies and their ability to adapt to changing environments. In addition, the selection for specific traits in domestic animals might have an additional impact on subjects' behavioural flexibility, but also their general learning performance, due to a re-allocation of resources towards parameters of productivity. To test whether animals bred for high productivity would experience a shift towards lower learning performance, we compared the performance of dwarf goats (not selected for production, 15 subjects) and dairy goats (selected for high milk yield, 18 subjects) in a visual discrimination learning and reversal learning task. Goats were tested individually in a test compartment and were rewarded by choosing either a white or a black cup presented by the experimenter on a sliding board behind a crate. Once they reached a designated learning criterion in the initial learning task, they were transferred to the reversal learning task. To increase the heterogeneity of our test sample, data was collected by two experimenters at two research stations following a similar protocol. Goats of both selection lines did not differ in the initial discrimination learning task in contrast to the subsequent reversal learning task. Dairy goats reached the learning criterion slower compared to dwarf goats (dairy goats = 9.18 sessions; dwarf goats = 7.74 sessions; *P* = 0.016). Our results may indicate that the selection for milk production might have affected behavioural flexibility in goats. These differences in adapting to changing environmental stimuli might have an impact on animal welfare e.g., when subjects have to adapt to new environments or changes in housing and management routines.

## Introduction

To survive, animals need to flexibly adapt to their environment (Shettleworth, 2010). Their ability to learn, and associated levels of behavioural flexibility, have been linked to many socio-ecological parameters, such as the diversity of food sources and habitats (Rosati, 2017), and to complex social group structures (Amici et al., 2008, 2009). Behavioural flexibility refers to the adaptive change in the behaviour of an animal, e.g., an animal's ability to learn a now reversed learning contingency. Another factor with the potential to impact behavioural flexibility, or learning ability in general, is artificial selection by humans, either by means of domestication (Price, 1999; Lindqvist and Jensen, 2009) or subsequent selection for specific production traits (Dudde et al., 2018). These differences can be of relevance in the context of various welfare-related issues in farm animals, such as adaptation to new environments or changes in housing and management routines, but remain relatively unexplored.

To assess behavioural flexibility, researchers often rely on the assessment of an individual's reversal learning ability (Berg, 1948). Although the test design is identical, reversal learning necessitates different and more complex cognitive mechanisms compared to simple discrimination learning (Diekamp et al., 1999). After meeting a certain learning criterion in an initial discrimination task, subjects will have to inhibit responses to the originally rewarded stimulus and to respond to a previously unrewarded stimulus in a reversal task. By using learning and reversal learning tasks, one can thus not only measure the general ability of an individual to learn, but also how flexible it can adapt its learned response.

During the course of domestication, with more stable food security and environmental conditions, selection pressure for improved learning performance and flexible adaptation to novel contexts might have been altered in domestic animals (Price, 1999). Research on learning performance comparing domestic species and their wild ancestors has come up with inconclusive results. While Lindqvist and Jensen found impaired spatial learning in domestic fowl compared to their non-domesticated counterparts (Lindqvist and Jensen, 2009), Gunther et al. showed that domestic guinea pigs (*Cavia porcellus*) learned an association faster than non-domesticated cavies (*Cavia aperea*), while both groups did not differ in their reversal learning performance (Brust and Guenther, 2015). Wolves (*Canis lupus*), in turn, differ in their behavioural flexibility compared to domestic dogs (*Canis lupus familiaris*) (Marshall-Pescini et al., 2015). However, the directionality of this difference was dependent on the task that was used (Marshall-Pescini et al., 2015).

Not only domestication, but also further selection and breeding of farm animals for high productivity can have an indirect impact on behavioural traits due to an assumed re-location of resources, according to the so-called Resource Allocation Theory (Beilharz et al., 1993). As animals bred for high performance may invest more resources into production traits and less in other biological processes, these changes might also potentially affect their ability to learn and their flexibility to adapt to new or variable environments. Indeed, selection for high productivity has already been found to have altered foraging and exploration behaviour in farm animals (Schütz and Jensen, 2001; Colpoys et al., 2014). In terms of potential impacts on mental processes, recent work on different production lines of laying hens found no, or contradictory, associations between selection for production and learning performance: hens that have been bred for higher egg yield were faster to reach a learning criterion in a visual discrimination task, compared to lines that have not been selected for high egg yield. However, both lines did not differ in their performance in the subsequent reversal learning task (i.e., do not show differences in behavioural flexibility; Dudde et al., 2018). Further investigations in the context of different production traits are necessary to assess potential associations between the selection for production traits, learning performance and behavioural flexibility.

In this study, we assess learning and behavioural flexibility in goats. Goats, as grassland foragers and prey animals, rely heavily on vision when navigating and have already shown to be able to master visual and spatial reversal tasks (Langbein et al., 2008; Meyer et al., 2012). Thus, a visual discrimination task and a subsequent reversal learning task were used to investigate whether goats not selected for production traits (dwarf goats) and goats selected for high milk yield (dairy goats) differ in their (reversal) learning ability. Based on the Resource Allocation Theory (Beilharz et al., 1993), we hypothesised dwarf goats outperforming dairy goats in the learning task, as well as in the reversal learning task.

## Methods

### Subjects, Housing, and General Procedure

To increase the heterogeneity of our sample, data was collected by two researchers at two research sites (Agroscope Tänikon in Ettenhausen, Switzerland, and the Research Institute for Farm Animal Biology in Dummerstorf, Germany) (Voelkl et al., 2018, 2020). 18 non-lactating female Nigerian dwarf goats (mean age ± SD; Ettenhausen: 364.4 ± 3.2 d, Dummerstorf: 361.7 ± 19.2 d at start of habituation) and 18 non-lactating female dairy goats (Ettenhausen: 339 ± 12.4 d, Dummerstorf: ~396 d at start of the initial visual discrimination task) participated in the experiment, that consisted of a visual discrimination task and a subsequent reversal learning task. The number of subjects for the current study was logistically limited due to their assignment for a specific treatment for a subsequent study (Nawroth et al., 2021). I.e., goats were a randomly chosen sub-sample of a larger group and were group-housed in 6 pens with 10 subjects each (of which three subjects per pen participated in this experiment and were thus assigned as one treatment group for a study that investigated the impact of test experience on individuals' performance in subsequent conceptually different tests) at both locations (Ettenhausen: *n* = 9 for dwarf and dairy goats each; Dummerstorf: *n* = 9 for dwarf and dairy goats each).

Dwarf goats for both locations were bred in Germany, Dummerstorf. Dairy goats were bred at different Swiss farms (Saanen and Chamois coloured goats) and one large German farm (Deutsche Edelziege). The Nigerian Dwarf goat is commonly kept as pet and zoo animal in Europe and not selected for productivity traits. The only selection aim in the Dummerstorf population was to avoid inbreeding. The potential milk yield of dwarf goats does likely not exceed 0.3 kg per day (Akinsoyinu et al., 1977). As it was common practise in Dummerstorf, dwarf goat kids stayed with their dams for 6 weeks before they were weaned. We used three of the most common high-producing dairy breeds in Switzerland and Germany (Saanen and Chamois coloured goats, Deutsche Edelziege). These animals had a potential milk yield of up to 3 kg per day (Vacca et al., 2018). In accordance with common practise in the dairy goat industry, the dairy goat kids had been separated from their dam shortly after birth and were artificially raised.

Initially, dwarf and dairy goats were housed in one large group pen (per selection line) at each location. At the age of 7–8 months, all goats were then moved to pens of 10 goats each. The total area of each dwarf goat pen was 14 m^2^ (~3.6 × 3.9 m), consisting of a deep-bedded straw area of 11 m^2^ (~2.8 × 3.9 m) and a 0.5 m elevated feeding place (1.4 m^2^). The total area of each dairy goat pen was 17.7 m^2^ (~3.9 × 4.55 m) consisting of a deep-bedded straw area of 13.4 m^2^ (~4.55 × 2.95 m) and a 0.65 m elevated feeding place (1.82 m^2^). Hay was provided behind a feeding fence at the feeding place twice a day at around 8 am and 4 pm in Ettenhausen and at around 7 am and 1 pm in Dummerstorf. Each pen had one watering place and a mineral supply. Additional structures in the straw-bedded area included a wooden bench (for dwarf: 2.3 m long, 0.5 m high, 0.5 m wide; for dairy: 2.4 m long, 0.6 m high, 0.62 m wide) along the wall of the pen and a round wooden table (0.8 m high, Ø 1.1 m) in the centre of the pen. Pens and handling regimes were kept as similar as possible at both locations.

**Figure 1:**
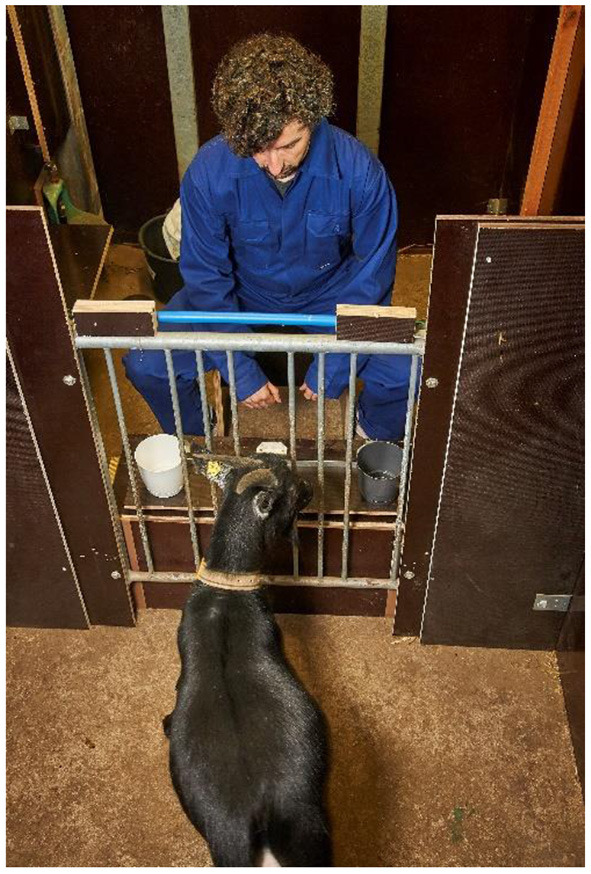
Example illustration of the test setup during the visual discrimination and reversal learning task. © Nordlicht/FBN.

For individual habituation, shaping, training, and testing, goats were physically and visually separated from their pen-mates in a test area (450 × 200 cm), but kept acoustic contact to their pen-mates that were located in an adjacent waiting area (600 × 200 cm). The experimenter sat in another adjacent compartment (150 × 200 cm) separated from the tested animal by a grate, allowing subjects to insert their snouts through the bars. A sliding board (60 × 20 cm) was placed on the experimenter side of the grate on a small table (105 × 40 cm) at a height of ~35 or 40 cm (for dwarf goats and dairy goats, respectively) in front of the grate (Figure 1). Subjects were not food restricted before testing. Goats were tested once a day (~between 9:00 and 12:00, with time of testing counterbalanced between subjects). To decrease potential experimenter biases, two experimenters (CN and KR) were alternating between each test session at both research sites.

### Habituation

Subjects were first introduced as a group (all subjects of each pen) for 2 days to the test arena and an adjacent waiting area for ~20 min per day. Subsequently, goats were introduced as pairs to the test arena. Each pair was provided ~10 pieces of reward (uncooked piece of pasta; positive reinforcement) over a period of 5 min *via* the sliding board (dwarf goats: 6 days; dairy goats: 4 days). Finally, subjects were habituated alone for 2 min, using the same procedure as for the paired habituation (dwarf goats: 13 days; dairy goats: 7 days). Habituation sessions were repeated until the individual showed no signs of arousal or stress during food delivery. All subjects reached the criterion to proceed with the shaping procedure.

### Shaping

Shaping was introduced to familiarise subjects to the test procedure and to train them how to indicate a choice. In shaping trials, one flat plastic bowl (brown, diameter 14 cm, height 2 cm) was located in the middle of the sliding board. In the first four trials of a shaping session, the experimenter put a food reward into the bowl and then pushed the platform towards the grate. If the animal put its nose through one of the middle gaps in the grate, it received a reward (positive reinforcement). This was repeated for six additional trials, but for these, the experimenter covered the bowl with a cup (light brown, diameter 12 cm, height 10.5 cm) before letting the subject make its choice. Shaping sessions were repeated until the individual showed no signs of arousal or stress during participation and instantly chose the baited position. If a subject did not make a choice within 60 s, a trial was repeated. If a trial had to be repeated twice, the session was terminated. Dwarf goats received a total of five shaping session, while dairy goats received a total of two sessions. Afterwards, all goats proceeded to the training. Dwarf and dairy goats differed in their emotional reactivity, so providing all goats with the same time of habituation and shaping might have led to different absolute levels of habituation/shaping for the two groups (Rosenberger et al., submitted)^1^. In addition, we chose a criterion on the group, rather than the individual level, as these goats were part of the treatment group (the group that received cognitive stimulation) for a subsequent study (Nawroth et al., 2021).

### Training

Training sessions were conducted for both selection lines and consisted of 10 trials each. All subjects received a maximum of two sessions per day. Two bowls were placed on the left and right side of the sliding board at a distance of 30 cm. The experimenter baited only one bowl in full view of the subject, then covered both bowls with identical cups (same size and colour as before) and pushed the board towards the grate. The subject made its choice by putting its snout through one of the outer left or right gaps in the grate (opposite the respective bowl) and the experimenter only delivered the reward if the subject was choosing the baited bowl. Each side was baited pseudorandomly five times per session and a maximum of five sessions were administered. Subjects were considered to have completed training when they achieved at least 8 out of 10 correct choices in two consecutive sessions (binomial test; *P* = 0.012).

### Visual Discrimination Task

All subjects participated first in the visual discrimination learning task. In this task, the experimenter (E) baited one of two different coloured cups (black or white, diameter 14.5 cm, height 12.0 cm) surreptitiously. The two different coloured cups were placed with the opening upwards on the left and right side of the sliding board at a distance of ~30 cm (Figure 1). Half of the subjects of each selection line were rewarded for choosing one particular coloured cup, whereas the other half was rewarded by choosing the other colour. After a presentation of ~2 s, E pushed the board towards the grate. The subject was able to make a choice by putting its snout through one of the outer left or right gaps in the grate (opposite the respective bowl) and, if the correct choice (e.g., the baited cup) was made, the goat obtained the reward. To avoid olfactory cueing, a piece of uncooked pasta was attached inside each cup. Each test session consisted of two initial motivation trials (one piece of pasta placed on either the left or right uncovered bowl on the board) at the beginning and 12 subsequent test trials as described above. The location of the baited cup was presented in a pseudo-randomised order across trials, but the baited cup was never positioned on the same side more than two trials in a row. After the first and second incorrect choice in each session, the goat had the opportunity to correct its choice: the non-rewarded cup was withdrawn, and the rewarded cup was kept on the board. After the goats chose the rewarded cup, it received the reward and a new trial started. This correction trial was still scored as incorrect. The inclusion of correction trials was done to prevent frustration and the development of side biases. Any subsequent error resulted in an immediate withdrawal of both cups, leaving the goat unrewarded. Subjects received as many sessions as needed to reach the specific learning criterion (10 out of 12 correct in two consecutive sessions).

### Reversal Learning Task

In the reversal learning task, the procedure was the same as in the initial discrimination task (including correction trials), except that in this task the previously unrewarded cup was rewarded, i.e., the reward contingencies were reversed. Again, we scored the number of sessions that goats needed to reach the learning criterion (10 out of 12 correct in two consecutive sessions).

Note that due to logistical reasons, each subject received a combined maximum number of 20 sessions for the visual discrimination and the reversal learning task. If a subject did not indicate a choice after 60 s, the trial was repeated. If the subject did not make a choice in the repeated trial, the session was terminated. After three consecutive terminated sessions, a subject was excluded from further testing. Consequently, two dwarf goats (Dummerstorf) were excluded from subsequent testing as they did not indicate a choice (visual discrimination learning session 1 and 8, respectively).

### Ethical Note

Animal care and all experimental procedures were in accordance with the ASAB/ABS Guidelines for the Use of Animals in Research (Association for the Study of Animal Behaviour). All procedures involving animal handling and treatment were approved by the Committee for Animal Use and Care of the Ministry of Agriculture, Environment and Consumer Protection of the federal state of Mecklenburg-Vorpommern, Germany (Ref. Nr. 7221.3-1.1-062/17) and by the Swiss Cantonal Veterinary Office, Thurgau (Approval No. TG04/17 – 29343). Housing facilities met the Swiss welfare requirements for farm animals. All measurements were non-invasive, and a session lasted no more than 10 min for each individual goat. If the goats had become stressed, e.g., were frequently vocalising and not paying attention to the test procedure during a test session, the test would have been stopped.

### Data Scoring and Analysis

A digital video camera (Ettenhausen: Sony HDR-CX240E; Dummerstorf: Panasonic HDC-SD60) was used to record all trials. We scored which cup (correct or incorrect) the test subject chose for each trial. A “correct” choice was scored if the subject chose the baited cup (i.e., by putting its snout through the respective gap in the grate). Fifteen dwarf goats and 18 dairy goats reached the learning criterion in the initial visual discrimination task. As one dairy goat took 10 sessions to finish in the visual discrimination task, it was subsequently not able to reach the criterion within the remaining 10 reversal learning sessions while all other goats did reach the criterion here, too. We assigned it to 12 reversal learning sessions, as this would have been the minimum number of total sessions for this task for this individual to reach the criterion.

To assess inter-observer reliability, 10% of the videos were coded by a second coder who was unfamiliar to the initial hypothesis. Inter-observer reliability for choice analysis showed a high level of agreement (Cohen's κ = 0.996, *P* < 0.001).

Statistical analyses were carried out in R v.3.6 (R Core Team, 2017). We scored the number of sessions a subject needed to reach the learning criterion [i.e., choosing correctly in 10 out of 12 trials (binomial test, *P* = 0.019) in two consecutive sessions] for both tasks. The number of sessions needed to reach this criterion was used as outcome variable and was analysed with a linear mixed-effects model (LMM) fit with gaussian family distribution (LMM; lmer function, lme4 library) (Pinheiro and Bates, 2000). Performances in the discrimination and reversal learning task were analysed separately in two models, because the underlying mechanisms to solve both tasks cannot be assumed to be identical (Diekamp et al., 1999). Both models included “Selection line” (factor with two levels: dwarf, dairy) and “Colour” of the rewarded container cup (factor with two levels: white, black) as well as their interaction as fixed factors. “Identity” of the goats nested in “Pen” (1–12) nested in “Location” (Ettenhausen, Dummerstorf) was included as a random factor to control for repeated measurements. For both models, we checked the residuals of the models graphically for normal distribution and homoscedasticity (simulateResiduals function, DHARMa library). *P*-values were calculated using parametric bootstrap methods (1,000 bootstrap samples, PBmodcomp function, pbkrtest library). *P*-values calculated with parametric bootstrap tests give the fraction of simulated likelihood ratio test (LRT) statistic values that are larger or equal to the observed LRT value. This test is more adequate than the raw LRT because it does not rely on large-sample asymptotic analysis and correctly takes the random-effects structure into account (Halekoh and Højsgaard, 2014).

Code and raw data are available at the Electronic Supplementary Material (ESM) and here: https://osf.io/tfmwc/.

## Results

### Training

Dwarf goats needed 2.94 ± 1.06 (mean ± SD) sessions to reach the criterion (Ettenhausen 2.50 ± 0.71 sessions; Dummerstorf: 3.33 ± 1.15 sessions), while dairy goats needed 2.78 ± 0.85 sessions to reach the criterion (Ettenhausen 2.78 ± 0.92 sessions; Dummerstorf: 2.78 ± 0.79 sessions). One subject (dwarf goat, Ettenhausen) did not reach the criterion and was excluded from further testing.

### Visual Discrimination Task

**Figure 2:**
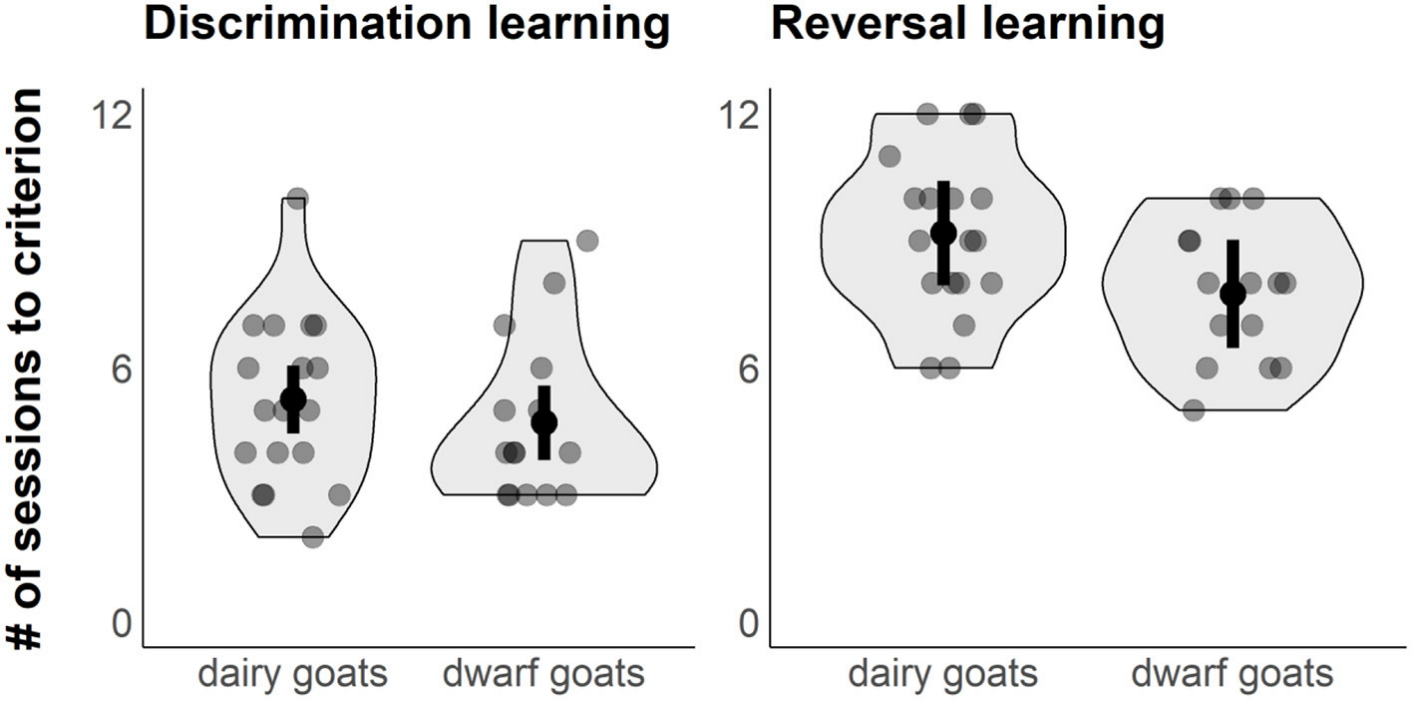
Violin plots with jittered raw data points (grey dots) showing the performance of dwarf and dairy goats in the initial visual discrimination learning task (left) and the reversal learning task (right). Figure shows also model estimates (black dots) and 95% CIs (error bar).

“Selection line” did not affect the number of sessions to reach the learning criterion in the initial visual discrimination task (est. ± CI: dwarf goats: 4.70 ± 0.43; dairy goats: 5.25 ± 0.39; *P* = 0.38, Figure 2). The colour of the rewarded cup (“Colour”) had an impact on the learning performance in the discrimination learning task, with the black stimulus being more easily learned than the white (est. ± CI: black stimulus: 4.05 ± 0.42; white stimulus: 5.89 ± 0.41; *P* = 0.005). There was no interaction for “Colour” and “Selection line” (*P* = 0.39). The variation (SD) explained by the random effects “Location” and “Pen” was < 0.001 for both.

### Reversal Learning Task

Dwarf goats needed fewer sessions compared to dairy goats to learn the reversal task (“Selection line,” est. ± CI: dwarf goats 7.74 ± 0.62, dairy goats 9.18 ± 0.60; *P* = 0.016, Figure 2). “Colour” did not affect performance in the reversal learning task (est. ± CI: black stimulus: 8.10 ± 0.62; white stimulus: 8.93 ± 0.61; *P* = 0.12). There was no interaction for “Colour” and “Selection line” (*P* = 0.77). Variation (SD) explained by the random effect “Pen” was < 0.001, while the variation for “Location” was 0.7.

## Discussion

To determine how selection for high productivity impacts general learning capacity and behavioural flexibility, we investigated the ability of dwarf (not selected for production traits) and dairy goats (selected for milk production) to solve a visual discrimination and a subsequent reversal learning task. While both groups performed similarly in the initial learning task, we found that dwarf goats performed better in the reversal task compared to dairy goat. Although other confounding factors need to be addressed in future research to rule out alternative explanation, our results provide first support for the hypothesis that selection for high productivity may be associated with decreased behavioural flexibility, but not initial learning performance, in goats.

The performance of goats in the initial learning task in this study (ranging from 2 to 10 session to reach the learning criterion, totalling in 24–120 trials) did not differ between both selection lines. At least for goats, and contrary to the Resource Allocation theory, the selection for high production does appear to not notably affect learning performance in a visual discrimination task. The goats' general performance in the task appears to be comparable to the performance of sanctuary goats tested with a similar design, but a slightly different protocol (8–96 trials to reach a learning criterion Nawroth and Prentice, 2017). When dwarf goats were presented with a four-choice visual discrimination task using an automated learning device with a screen (i.e., presented 2D stimuli), they needed 180–620 trials until they reached a designated learning criteria (Langbein et al., 2007), indicating that providing live stimuli appears to enhance learning by making the set up less arbitrary (O'Hara et al., 2015). Many other factors, ranging from different thresholds regarding a learning criterion to differences in the design of these visual discrimination tasks have likely also contributed to this variation and make rigorous comparisons not feasible. Future research should aim to better standardise test protocols in order to increase comparability of results.

In the reversal task dwarf goats outperformed dairy goats which lends support to the hypothesis that the Resource Allocation theory can also be applied to some cognitive functions in farm animals (Beilharz et al., 1993). However, Dudde et al. did not find such a relationship in their reversal learning task (Dudde et al., 2018) between two lines of hens that have been bred for higher egg yield compared to two lines that have not been selected for high egg yield. One reason for a lack of this pattern could be a relatively high drop-out rate in the lines not selected for high egg yield (40 and 60% of individuals, respectively, at the start of the initial learning task), which could have led to a biassed overall comparison of both groups. In our study, the only drop-outs were as well from the line that was not selected for high performance (dwarf goats) but with a moderate rate only (~17%). However, the difference in performance between dwarf and dairy goats in the reversal learning was relatively small, making it difficult to infer the biological meaningfulness of the observed effect when applied to other contexts in a farm setting in which learning might occur (e.g., locating and remembering new drinker and feeder positions after transfer to new environments).

Although both selection lines were handled in a similar manner at the research facilities, early ontogenetic factors could have also played a role and might explain the observed differences. As it is common for the dairy industry, the dairy goats used in this study had been separated from their dam right after birth. In contrast, dwarf goats had been allowed to stay with their mothers for 6 weeks. Research has shown that early separation from the dam and rearing by humans increased tameness scores in goats (Lyons et al., 1988). If this would have been the case with our subjects, we would have expected a better performance in dairy compared to dwarf goats, as tamer individuals exhibit less stress during handling, and stress, in turn, can affect memory and learning (Mendl, 1999; Valenchon et al., 2013; Brajon et al., 2016). In addition, differences in sociality, rather than behavioural flexibility, might account for the detected differences. Subjects of both selection lines might differ in how they cope with isolation during the test situation, which in turn could have impacted reversal learning performance. Another factor limiting the general applicability of our results is that we could only test one breed of the non-production line, heightening the risk that the found differences in the reversal learning task might be due to specifics of this breed (or even population), rather than a result of non-selection for production.

Interestingly, goats were faster to learn a colour association when the stimulus was a black cup rather than a white cup in the learning, but not reversal learning task. Goats might have been biassed due to the setup of the training sessions. Although light brown cups have been used during training, we cannot exclude the possibility that goats transferred this association to the darker cup in the test trials. So other settings, such as the colour and shape of the stimulus, can impact learning performance and future designs should aim to strongly adhere to species-specific limitations regarding visual acuity and colour discrimination ability. Although in our study we found no interaction with learning performance, this finding also highlights the need for a balanced presentation of the stimuli and/or stimulus preference tests prior to the test situation.

## Conclusions

Differences in behavioural flexibility could affect the ability of specific selection lines to adapt to new environments or changes in housing and management routines and thus might be relevant for the welfare of domestic animals. Our results provide first support to the hypothesis that selection for high milk yield in goats might be associated with decreased behavioural flexibility.

## Data Availability Statement

Code and raw data are available in the Supplementary Material and in a public repository here: https://osf.io/tfmwc/.

## Ethics Statement

All procedures involving animal handling and treatment were approved by the Committee for Animal Use and Care of the Ministry of Agriculture, Environment and Consumer Protection of the federal state of Mecklenburg-Vorpommern, Germany (Ref. Nr. 7221.3-1.1-062/17) and by the Swiss Cantonal Veterinary Office, Thurgau (Approval No. TG04/17 /17rova). Written informed consent was obtained from the individual(s) for the publication of any potentially identifiable images or data included in this article.

## Author Contributions

CN, NK, and JL conceptualised the study. CN and KR collected the data. CN analysed the data and wrote the main part of the manuscript. All authors provided critical feedback on the manuscript and gave approval for submission.

## Funding

This work was supported by grants from the Deutsche Forschungsgemeinschaft (DFG, LA 1187/6-1 to JL), Swiss National Fond (SNF, 310030E-170537 to NK), and Open Access Fund of the Research Institute for Farm Animal Biology.

## Conflict of Interest

The authors declare that the research was conducted in the absence of any commercial or financial relationships that could be construed as a potential conflict of interest.

## Publisher's Note

All claims expressed in this article are solely those of the authors and do not necessarily represent those of their affiliated organizations, or those of the publisher, the editors and the reviewers. Any product that may be evaluated in this article, or claim that may be made by its manufacturer, is not guaranteed or endorsed by the publisher.

## Footnotes

^1^Rosenberger, K., Simmler, M., Nawroth, C., Langbein, J., and Keil, N. (2021). *Reactivity of Domesticated Goats Towards Various Stressors Following Long-Term Cognitive Test Exposure* (submitted).
