# Supplementary material for: Goats (Capra hircus) From Different Selection Lines Differ in Their Behavioural Flexibility
Source: Front Psychol. 2022 Feb 1;12:796464. doi: 10.3389/fpsyg.2021.796464 (PMC8844551; doi:10.3389/fpsyg.2021.796464)
Supplement: Supplementary file 1 [file Table_1.DOCX]

####################################################################

####################################################################

## Paper: Goats (Capra hircus) with different selection ##

## objectives differ in their behavioural flexibility ##

## ##

## Authors: C. Nawroth, K. Rosenberger, N. Keil, J. Langbein ##

## ##

## Code Author: CN ##

## ##

## Version: 1.0 ##

## ##

## Software: R 4.0.2 ##

####################################################################

####################################################################

###################

## Load packages ##

###################

library(lme4)

library(tidyverse)

library(cowplot)

library(ggpubr)

library(gghalves)

library(pbkrtest)

library(DHARMa)

library(ggplot2)

library(scales)

library(see)

library(effects)

options(scipen=999)

#################

## Import Data ##

#################

dat.df <- read.delim("ESM_learning.csv", header = TRUE, sep= ',')

str(dat.df)

#dat.df$line<-as.factor(dat.df$line)

#dat.df$colour<-as.factor(dat.df$colour)

#dat.df$location<-as.factor(dat.df$location)

#dat.df$pen<-as.factor(dat.df$pen)

#str(dat.df)

##############

## Learning ##

##############

learn_full <- lmer(sessionsL ~ 1 + line * colour + (1|location/pen), data = dat.df, na.action=na.exclude, REML=FALSE)

learn_1 <- lmer(sessionsL ~ 1 + line + colour + (1|location/pen), data = dat.df, na.action=na.exclude, REML=FALSE)

learn_2 <- lmer(sessionsL ~ 1 + line + (1|location/pen), data = dat.df, na.action=na.exclude, REML=FALSE)

learn_3 <- lmer(sessionsL ~ 1 + colour + (1|location/pen), data = dat.df, na.action=na.exclude, REML=FALSE)

#Model assumptions

simulationOutput <- simulateResiduals(fittedModel = learn_1, n = 250)

plot(simulationOutput)

testResiduals(simulationOutput)

#Bootstrapping

set.seed(1000)

learn_bs_interaction <- PBmodcomp(learn_full, learn_1)

learn_bs_colourL <- PBmodcomp(learn_1, learn_2)

learn_bs_line <- PBmodcomp(learn_1, learn_3)

summary(learn_bs_interaction)

summary(learn_bs_colourL)

summary(learn_bs_line)

#Covariance by random factors

VarCorr(learn_1)

#Calculating CIs

CI_learn_all <- allEffects(learn_1)

as.data.frame(CI_learn_all)

CI_learn_line <- Effect("line", learn_1)

CI_learn_line.df <- data.frame(CI_learn_line)

CI_learn_colour <- Effect("colour", learn_1)

CI_learn_colour.df <- data.frame(CI_learn_colour)

##############

## Reversal ##

##############

reversal_full <- lmer(sessionsR ~ 1 + line * colour + (1|location/pen), data = dat.df, na.action=na.exclude, REML=FALSE)

reversal_1 <- lmer(sessionsR ~ 1 + line + colour + (1|location/pen), data = dat.df, na.action=na.exclude, REML=FALSE)

reversal_2 <- lmer(sessionsR ~ 1 + line + (1|location/pen), data = dat.df, na.action=na.exclude, REML=FALSE)

reversal_3 <- lmer(sessionsR ~ 1 + colour + (1|location/pen), data = dat.df, na.action=na.exclude, REML=FALSE)

#Model assumptions

simulationOutput <- simulateResiduals(fittedModel = reversal_1, n = 250)

plot(simulationOutput)

testResiduals(simulationOutput)

#Bootstrapping

reversal_bs_interaction <- PBmodcomp(reversal_full, reversal_1)

reversal_bs_colourR <- PBmodcomp(reversal_1, reversal_2)

reversal_bs_line <- PBmodcomp(reversal_1, reversal_3)

summary(reversal_bs_interaction)

summary(reversal_bs_colourR)

summary(reversal_bs_line)

#Covariance by random factors

VarCorr(reversal_full)

#Calculating CIs

CI_reversal_all <- allEffects(reversal_1)

as.data.frame(CI_reversal_all)

CI_reversal_line <- Effect("line", reversal_1)

CI_reversal_line.df <- data.frame(CI_reversal_line)

CI_reversal_colour <- Effect("colour", reversal_1)

CI_reversal_colour.df <- data.frame(CI_reversal_colour)

############

## Figure ##

############

#dat.df$breed <- factor(dat.df$breed, levels = c("Dwarf", "Dairy"))

#dat.df$location <- factor(dat.df$location, levels = c("Ettenhausen", "Dummerstorf"))

selectionline <- c("dairy goats", "dwarf goats")

fig1 = ggplot(CI_learn_line.df, aes(x=line, y=fit)) +

scale_x_discrete(labels=selectionline) +

scale_fill_viridis_d() +

geom_violin(data=dat.df, aes(x=line, y=sessionsL), alpha = 0.4, colour="black", fill="#CCCCCC") +

geom_jitter(data=dat.df, aes(x=line, y=sessionsL), size=5, position=position_jitter(h=0.00,w=0.20), alpha = 0.4, show.legend = F) +

geom_point(size = 6) +

geom_errorbar(aes(ymax=lower, ymin=upper, width = .0), size=3) +

theme_modern() +

#xlab("Selection line") +

scale_y_continuous(breaks=seq(0,12,6), limits=c(0,12)) +

ylab("# of sessions to criterion") +

ggtitle("Discrimination learning") +

theme(plot.title = element_text(face="bold", size=24),

axis.title.x = element_blank(),

axis.text.x = element_text(size=20),

axis.title.y = element_text(face="bold", size=24),

axis.text.y = element_text(size=20),

strip.text = element_text(size = 20))

fig2 = ggplot(CI_reversal_line.df, aes(x=line, y=fit)) +

scale_x_discrete(labels=selectionline) +

scale_fill_viridis_d() +

geom_violin(data=dat.df, aes(x=line, y=sessionsR), alpha = 0.4, colour="black", fill="#CCCCCC") +

geom_jitter(data=dat.df, aes(x=line, y=sessionsR), size=5, position=position_jitter(h=0.00,w=0.20), alpha = 0.4, show.legend = F) +

geom_point(size = 6) +

geom_errorbar(aes(ymax=lower, ymin=upper, width = .0), size=3) +

theme_modern() +

scale_y_continuous(breaks=seq(0,12,6), limits=c(0,12)) +

#xlab("Selection line") +

#ylab("# of sessions to criterion") +

ggtitle("Reversal learning") +

theme(plot.title = element_text(face="bold", size=24),

axis.title.x = element_blank(),

axis.text.x = element_text(size=20),

axis.title.y = element_blank(),

axis.text.y = element_text(size=20),

strip.text = element_text(size = 20))

ggarrange(fig1, fig2,

#labels=c("A", "B"),

ncol=2, nrow=1)

ggsave("fig_results.png", units = "in", width = 10, height = 5, dpi = 300)
