# Supplementary material for: Goats (Capra hircus) From Different Selection Lines Differ in Their Behavioural Flexibility
Source: Front Psychol. 2022 Feb 1;12:796464. doi: 10.3389/fpsyg.2021.796464 (PMC8844551; doi:10.3389/fpsyg.2021.796464)
Supplement: Supplementary file 2 [file Table_2.pdf]

```
####################################################################
####################################################################
Paper:   Goats (Capra hircus) with different selection objectives differ in their behavioural flexibility
Authors: C. Nawroth, K. Rosenberger, N. Keil, J. Langbein
Code Author: CN
####################################################################
####################################################################

###################
## Load packages ##
###################

library(lme4)
library(tidyverse)
library(cowplot)
library(ggpubr)
library(gghalves)
library(pbkrtest)
library(DHARMa)
library(ggplot2)
library(scales)
library(see)
library(effects)
options(scipen=999)

#################
## Import Data ##
#################

dat.df <- read.delim("ESM_learning.csv", header = TRUE, sep= ',')

str(dat.df)
```

```
#dat.df$line<-as.factor(dat.df$line)

#dat.df$colour<-as.factor(dat.df$colour)

#dat.df$location<-as.factor(dat.df$location)

#dat.df$pen<-as.factor(dat.df$pen)

#str(dat.df)


##############

## Learning ##

##############


learn_full <- lmer(sessionsL ~ 1 + line * colour + (1|location/pen), data = dat.df,
na.action=na.exclude, REML=FALSE)

learn_1 <- lmer(sessionsL ~ 1 + line + colour + (1|location/pen), data = dat.df, na.action=na.exclude,
REML=FALSE)

learn_2 <- lmer(sessionsL ~ 1 + line + (1|location/pen), data = dat.df, na.action=na.exclude,
REML=FALSE)

learn_3 <- lmer(sessionsL ~ 1 + colour + (1|location/pen), data = dat.df, na.action=na.exclude,
REML=FALSE)


#Model assumptions

simulationOutput <- simulateResiduals(fittedModel = learn_1, n = 250)

plot(simulationOutput)

testResiduals(simulationOutput)


#Bootstrapping

set.seed(1000)

learn_bs_interaction <- PBmodcomp(learn_full, learn_1)

learn_bs_colourL <- PBmodcomp(learn_1, learn_2)

learn_bs_line <- PBmodcomp(learn_1, learn_3)

summary(learn_bs_interaction)

summary(learn_bs_colourL)

summary(learn_bs_line)
```

```
#Covariance by random factors
VarCorr(learn_1)

#Calculating CIs
CI_learn_all <- allEffects(learn_1)
as.data.frame(CI_learn_all)

CI_learn_line <- Effect("line", learn_1)
CI_learn_line.df <- data.frame(CI_learn_line)

CI_learn_colour <- Effect("colour", learn_1)
CI_learn_colour.df <- data.frame(CI_learn_colour)

##############
## Reversal ##
##############

reversal_full <- lmer(sessionsR ~ 1 + line * colour + (1|location/pen), data = dat.df, na.action=na.exclude, REML=FALSE)
reversal_1 <- lmer(sessionsR ~ 1 + line + colour + (1|location/pen), data = dat.df, na.action=na.exclude, REML=FALSE)
reversal_2 <- lmer(sessionsR ~ 1 + line + (1|location/pen), data = dat.df, na.action=na.exclude, REML=FALSE)
reversal_3 <- lmer(sessionsR ~ 1 + colour + (1|location/pen), data = dat.df, na.action=na.exclude, REML=FALSE)

#Model assumptions
simulationOutput <- simulateResiduals(fittedModel = reversal_1, n = 250)
plot(simulationOutput)
testResiduals(simulationOutput)

#Bootstrapping
reversal_bs_interaction <- PBmodcomp(reversal_full, reversal_1)
```

```
reversal_bs_colourR <- PBmodcomp(reversal_1, reversal_2)
reversal_bs_line <- PBmodcomp(reversal_1, reversal_3)
summary(reversal_bs_interaction)
summary(reversal_bs_colourR)
summary(reversal_bs_line)

#Covariance by random factors
VarCorr(reversal_full)

#Calculating CIs
CI_reversal_all <- allEffects(reversal_1)
as.data.frame(CI_reversal_all)

CI_reversal_line <- Effect("line", reversal_1)
CI_reversal_line.df <- data.frame(CI_reversal_line)

CI_reversal_colour <- Effect("colour", reversal_1)
CI_reversal_colour.df <- data.frame(CI_reversal_colour)



############
## Figure ##
############

#dat.df$breed <- factor(dat.df$breed, levels = c("Dwarf", "Dairy"))
#dat.df$location <- factor(dat.df$location, levels = c("Ettenhausen", "Dummerstorf"))
selectionline <- c("dairy goats", "dwarf goats")

fig1 = ggplot(CI_learn_line.df, aes(x=line, y=fit)) +
  scale_x_discrete(labels=selectionline) +
  scale_fill_viridis_d() +
  geom_violin(data=dat.df, aes(x=line, y=sessionsL), alpha = 0.4, colour="black", fill="#CCCCCC") +
```

```
  geom_jitter(data=dat.df, aes(x=line, y=sessionsL), size=5, position=position_jitter(h=0.00,w=0.20), alpha = 0.4, show.legend = F) +
  geom_point(size = 6) +
  geom_errorbar(aes(ymax=lower, ymin=upper, width = .0), size=3) +
  theme_modern() +
  #xlab("Selection line") +
  scale_y_continuous(breaks=seq(0,12,6), limits=c(0,12)) +
  ylab("# of sessions to criterion") +
  ggtitle("Discrimination learning") +
  theme(plot.title = element_text(face="bold", size=24),
        axis.title.x = element_blank(),
        axis.text.x = element_text(size=20),
        axis.title.y = element_text(face="bold", size=24),
        axis.text.y = element_text(size=20),
        strip.text = element_text(size = 20))

fig2 = ggplot(CI_reversal_line.df, aes(x=line, y=fit)) +
  scale_x_discrete(labels=selectionline) +
  scale_fill_viridis_d() +
  geom_violin(data=dat.df, aes(x=line, y=sessionsR), alpha = 0.4, colour="black", fill="#CCCCCC") +
  geom_jitter(data=dat.df, aes(x=line, y=sessionsR), size=5, position=position_jitter(h=0.00,w=0.20), alpha = 0.4, show.legend = F) +
  geom_point(size = 6) +
  geom_errorbar(aes(ymax=lower, ymin=upper, width = .0), size=3) +
  theme_modern() +
  scale_y_continuous(breaks=seq(0,12,6), limits=c(0,12)) +
  #xlab("Selection line") +
  #ylab("# of sessions to criterion") +
  ggtitle("Reversal learning") +
  theme(plot.title = element_text(face="bold", size=24),
        axis.title.x = element_blank(),
        axis.text.x = element_text(size=20),
```

```
    axis.title.y = element_blank(),
    axis.text.y = element_text(size=20),
    strip.text = element_text(size = 20))

ggarrange(fig1, fig2,
     #labels=c("A", "B"),
     ncol=2, nrow=1)

ggsave("fig_results.png", units = "in", width = 10, height = 5, dpi = 300)
```
